# The Book World of Medicine and Science

**Published:** 1903-01-24

**Authors:** 


### The Book World of Medicine and Science.

Practical Physiology. By A. P. Beddard, Guy's Hospital; J. S. Edkins, St. Bartholomew's Hospital; Leonard Hill, London Hospital; J. J. R. Macleod, London Hospital; and M. S. Pembery, Guy's Hospital. (London: Arnold. 1902. Pp. 495, with numerous diagrams and tracings. Price 15s. net.)

The appearance of this admirable manual of laboratory practice in physiology, prepared by teachers at leading hospital schools in London, makes us reflect a little on the burden that rests on the shoulders of the modern medical student. In the old days, the student at the hospitals set his face resolutely from the beginning towards medicine. He entered the wards at once, and his work in the school was strictly subservient to the medical goal. The lecturers on anatomy, chemistry, physiology, and so forth were physicians and surgeons practising at the hospital, and all that he learned had its strictly medical and surgical application. The five authors of this volume are all qualified medical men, it is true, but at least three of them have abandoned medicine for science as a pursuit, and the volume very plainly shows that knowledge of physiology as a science and not merely as a preparation for another study was the dominant idea in their scheme. No doubt a time may come when medicine will be a pure deduction from anatomy, physiology and pathology, and in those days the best practitioner will be he who is best trained in the fundamental sciences. But at present this is far off, and the average student has to acquire his medical knowledge on principles drawn from medical experience and with only a comparatively remote relation to abstract science. We should have preferred to see in a volume obviously directed to the medical student and to the teacher of medical students, the principles of physiology more directly oriented to medical pursuits, less technical, and less elaborate. Apart from such *a priori* criticism, we have nothing but praise for the volume. The very elaborate appliances and experiments of modern physiology are described and explained fully and lucidly, and a great deal of new work, much of it done by the authors themselves, has found its appropriate place. Dr. Hill's sections on the blood, blood-pressures and respiration are very much in advance of the similar sections in other works. Dr. Pembery has carried on the muscle-nerve studies for which the Oxford laboratory under Sir J. Burdon Sanderson was so famous, and has added much new pharmacological matter. Dr. Macleod is very clear and very modern in his discussions of the difficult problems of physiological chemistry. If the volume had done no more, it has at least served to show that physiological teaching at the great hospital schools is on a level with that at the modern universities. For students above the average, and for those who are engaged or hope to be engaged in the advancement of medicine rather than its practice, it should prove of the highest service, and it shows that so far as physiology is concerned, the medical schools are fully worthy of their place as constituent colleges of the University of London.

Avenues to Health. By Eustace H. Miles, M.A. (London: Swan, Sonnenschein and Co., Limited. 1902. Price 4s. 6d.)

Mr. Eustace Miles has not only won distinction in the athletic world and made for himself a position at Cambridge but has also shown exceptional fertility in the making of books. Already he has issued some fifteen works, several of considerable size. His "Avenues to Health" seeks to afford a knowledge of means whereby a sound and vigorous life may be secured. Without undue dogmatism he urges the necessity for testing various methods, since it is "personal experience which alone can give us certain knowledge." And thus we are incited to adopt "the cheapest and easiest and purest means," such as "simpler foods and fewer meals, fresh air and the habit of correct breathing, sensible clothing, light and colour, heat, water, the habits of correct bodily position, games and athletics adapted to city life, brisk feet-movement exercises, muscular relaxing and the storing of energy, music, imagination and 'self-suggestion,' gradual training of sensation and observation, intellect, emotion and desire, will and action," and so on. It cannot be denied that these 438 pages, with their 64 chapters, contain much that is wearisome to the flesh and irritating to the mind. A layman, devoid of scientific principles and ignorant of medical methods of investigation, is only too frequently, however honest in purpose and diligent in experiment, a victim of quackery and charlatanism, and while this book will no doubt prove suggestive to intelligent minds willing to wade through redundances, repetitions and platitudes galore, it is certainly not a desirable manual for the average man. Mr. Miles quotes Tennyson to the effect that "knowledge comes, but wisdom lingers," and most unprejudiced minds after reading this, in many ways, remarkable book, will, we think, agree that, however well-intentioned such a work may be, it is hardly likely to assist in the development of "a sound understanding."

